# Active labour market policies in emerging adulthood may act as a protective factor against future depressiveness: an analysis of the long-term trajectories of depressive symptoms in the Northern Swedish Cohort

**DOI:** 10.3389/fpubh.2024.1345034

**Published:** 2024-04-09

**Authors:** Pekka Virtanen, Tapio Nummi, Hugo Westerlund, Per-Olof Östergren, Urban Janlert, Anne Hammarström

**Affiliations:** ^1^Faculty of Social Sciences, Tampere University, Tampere, Finland; ^2^Karolinska Institutet, Stockholm, Sweden; ^3^Faculty of Information Technology and Communication Sciences, Tampere University, Tampere, Finland; ^4^Department of Psychology, Stress Research Institute, Stockholm University, Stockholm, Sweden; ^5^Social Medicine and Global Health, Department of Clinical Sciences in Malmö, Lund University, Lund, Sweden; ^6^Department of Epidemiology and Global Health, Umeå University, Umeå, Sweden

**Keywords:** cohort study, life-course epidemiology, trajectory analysis, Sweden, mental health

## Abstract

**Introduction:**

Drawing upon the framework of life course epidemiology, this study aligns with research on the mental health consequences of significant social transitions during early adulthood. The focus is on the variation in initial labour market attachment and the development of depressiveness, assuming that a firm attachment is associated with decreasing depressiveness.

**Methods:**

The baseline investigation of the studied cohort (*n* = 1,001) took place during their final year of compulsory schooling at age 16. Follow-up surveys were conducted at ages 18, 21, 30, and 43. Depressiveness was measured with a five-item score. Multiple trajectory analysis, incorporating five labour market statuses observed over seven half-year periods from ages 18 to 21, was employed to categorize the cohort into six distinct groups. Among these, ‘All-time education,’ ‘From education to employment,’ ‘Education and employment,’ and ‘From employment to education’ were considered to demonstrate firm labour market attachment. Meanwhile, ‘Active labour market policy’ and ‘Unemployment’ represented less firm attachment.

**Results:**

The trajectory of depressive symptoms among the total cohort from age 16 to age 43 exhibited a ‘broken stick’ pattern, reaching its lowest point at age 21. This pattern was evident in all groups classified as having a firm attachment. A substantial decrease in depressiveness was also observed in the relatively weakly attached ‘Active labour market policy’ group, whereas no ‘broken stick’ pattern emerged in the ‘Unemployment’ group. The disparities in the levels of depressiveness observed at age 21 remained relatively stable across the measurements at ages 30 and 43.

**Discussion:**

The results were as expected, except for the observed improvement in mental health within the ‘Active labour market policy’ group. Supported labour market attachment during emerging adulthood can enhance mental well-being similarly to regular mainstream attachment. In terms of policy recommendations, the consistently high levels of depressiveness within the ‘Unemployment’ group underscore the importance of reducing long-term and repeated unemployment in young age. The findings regarding the ‘Active labour market policy’ provide evidence of the intervention’s benefits. While the primary goal of these measures is to create jobs for the unemployed, they also include elements that contribute to participants’ mental health.

## Introduction

Transitioning into adulthood involves assuming various social roles, among which establishing oneself as a participant in the labor market is crucial (1, 2). This study zeroes in on the age of 21, a pivotal period where attachment to the labor market is still in its formative stages, and examines its correlation with mental health development. The data utilized are sourced from the Northern Swedish Cohort (NoSCo) (3). Previous research on this cohort (4, 5) has revealed a significant turning point in the trajectory of mental health at age 21: after experiencing a steep decline from age 16 to 21, the cohort’s mental health symptoms tend to increase steadily up to age 43.

From a policy standpoint, understanding the underlying causes of such a ‘broken stick’ pattern is of paramount importance (4, see 6). This paper delves into the impact of various labor market positions and active labour market policy measures before age 21 on the shift in the development of depressive symptoms at age 21.

Drawing upon the framework of life course epidemiology (7), this study examines exposures to significant social transitions during early adulthood. This period has been recognized as particularly sensitive to experiencing both adverse and advantageous effects of these transitions on mental well-being:

*“The transition from adolescence to adulthood is one of the most critical of normative life transitions because it typically involves pervasive and often simultaneous contextual and social role changes. However, the question remains as to whether this transition is equally powerful in influencing the course of psychopathology and mental health. To what extent does this global transition (and all of the* var*ious developmental transitions embedded within it) contribute to continuity and discontinuity in psychopathology and mental health?”* [(8), pp. 799–800]

The decrease in depressiveness from age 16 to age 21 can be attributed to a smooth and successful entry into the labour market after shorter or longer post-basic education. Conversely, challenges in the labour market attachment (henceforth ‘LMA’), particularly unemployment (9), have a detrimental effect on mental health. A pertinent policy question is whether LMA drives the broken stick pattern of depressive symptoms. Moreover, it is important to know if various conventional pathways of entering the labour market are equally associated to the decrease of depressiveness during emerging adulthood. There is a lack of studies that consider, instead of focusing solely on unemployment, exposures to various sequences of labour market positions. Regarding outcomes, research focusing on the long-term development of mental health is scarce.

The outcome of the current study focuses on the development of depressive symptoms in a population cohort from age 16 to 43, revealing a broken stick pattern with a notable turning point at age 21. This decline during early adulthood is consistent with findings in other studies (10–12), although some studies report contrasting trends (13, 14). In later adulthood, the Norwegian HUNT study (15) observed a continuous linear increase in depressive symptoms from the 20–29 age group to the 80–89 age group, even after accounting for a wide range of potential risk factors (16). In contrast, Australian studies have demonstrated a systematic decrease in symptoms from the 18–34 age group to those over 65 (17) and from the 18–29 age group to the 60–69 age group (18). While the evidence is not entirely consistent, it suggests the existence of a pivotal point in the prevalence of depression during the transition from emerging adulthood to early adulthood. However, earlier research aimed at pinpointing this turning point is lacking.

Expanding upon the ‘labour market attachment’ concept previously explored in research within the NoSCo (19), the present study introducess the concept of ‘Initial Labor Market Attachment’ (ILMA). ILMA focuses on the phase of the life course that follows the completion of compulsory basic school education. During this phase, individuals engage in simultaneous and consecutive episodes aimed at acquiring additional knowledge and skills with the goal of establishing and stabilizing their occupational or professional labour market status. This concept differs from the traditional notion of ‘school-to-work transition’, which overlooks the multitude and complexity of pathways young people take as they enter the workforce (20), and from the stigmatizing ‘Not in Employment, Education, or Training’ (NEET) concept (21–23).

In addition to various combinations of studying and working, ILMA also encompasses periods of unemployment and times when individuals stay outside the labour market for various reasons. Consequently, it allows for a more detailed analysis of the associated development of mental health implied by earlier studies in the NoSCo dataset (24–27). The aim of this paper is to test the hypothesis that a firm labour market attachment can explain the decrease of depressive symptoms from age 16 to age 21.

## Methods

### Locality

The study involves participants from a medium-sized industrial town in Northern Sweden, comprising around 80,000 inhabitants. This town serves as a representative sample of similar-sized industrial towns in Sweden, reflecting comparable sociodemographic factors and labour market conditions. Notably, at the outset of the study in 1981, the town exhibited double the average unemployment rate. Consequently, specific local policy measures were introduced to address this issue. By international standards, during the 1980s Sweden implemented a notably active labour market policy, particularly aimed at assisting young people. The local municipalities have since then responsibilities for providing activities for pupils in NEET after leaving compulsory school (up to age 18). Various forms of youth programs were introduced during the early 1980ies as introductions into the labour market, like work-place training 20 to 40 h a week during several months and certain payment was provided. This Swedish model has been worldwide acknowledged for offering universalism, comparatively generous replacement rates, and extensive welfare services.

### Population

The Northern Swedish Cohort (NoSCo) comprises all pupils (*n* = 1,083, 53% male) who completed their final year of compulsory schooling in 1981 in a municipality in Northern Sweden. They were 16 years old at the baseline investigation, and follow-up assessments were conducted at ages 18, 21, 30, and 43. Questionnaires were administered at each assessment point, and a comprehensive description of data collection items and procedures is available in a cohort profile (3). The analytical sample for this study includes 1,001 respondents (482 women and 519 men) from NoSCo.

Depressiveness at ages 16, 21, 30, and 43 was assessed using a previously validated index of six self-reported symptoms experienced in the preceding 12 months: poor appetite, general tiredness, concentration difficulties, sleep problems, feeling down/sad, and feeling dejected about the future (28). A continuous scale ranging from 0 to 2 was constructed and utilized, with higher scores indicating more severe depressive symptoms.

The variable ‘Initial Labour Market Attachment’ (ILMA) was derived from data collected in the survey at age 21, which included a matrix of 12 status alternatives and 7 semesters, spanning from spring 1983 to spring 1986 (ages 18–21). Respondents were instructed to mark all the statuses (one or more) they had experienced during each semester. These status alternatives were categorized into five variables:

1. ‘Education’ (including gymnasium, university, other education).
2. ‘Work’ (including full-time, part-time, occasional jobs).
3. ‘Active Labour Market Policy (ALMP)’ (including educational and vocational activities such as subsidised employment).
4. ‘Unemployment’.
5. ‘Other’ (including travelling, parental leave etc.).

### Statistics

The data on ILMA, which consisted of seven repeated measurements of five binary statuses (yes or no), was analysed using latent class analysis of multiple responses (29, 30). Following initial exploratory analyses with different polynomial degrees, the second-degree polynomial on age was determined to be the most appropriate model for each of the five binary positions. This model provided the necessary flexibility to account for non-linear developments within the subgroups. The final model was estimated using the Flexmix package in R (31).

The Bayesian Information Criteria (BIC) indicated that the nine-class solution was the best fit. These classes and their disposition into six groups are presented in detail in the Supplementary File. The groups are:

1. ‘All-time education’
2. ‘From education to employment’
3. ‘Education and employment’
4. ‘From employment to education’
5. ‘Active labour market policy’ (ALMP)
6. ‘Unemployment’

In terms of the firmness of the ILMA, groups 1, 2, 3 and 4 can be considered to represent different types of firm attachment, while group 5 represents a less firm attachment, and group 6 represents the least firm attachment.

The examination of mean depressive symptom scores at different ages revealed the highest value (0.48) at age 16, which decreased to 0.39 by age 21 but then increased again in middle age (0.45 at age 30 and 0.44 at age 43). Modelling this type of development with a first or second-degree polynomial may not adequately capture the shape of individual development curves for trajectory analysis. Conversely, more complex models can lead to overfitting and unstable estimates. Our approach was to use the ‘broken stick’ model, a continuous curve that joins two linear trend models at a specific ‘knot point,’ which in this case was set at age 21 (6). This model provides a continuous curve by connecting the linear trend model for ages 16 to 21 with the model for ages 21, 30, and 42.

Likelihood ratio tests were employed to assess the significance of the differences between the broken stick model and the linear model, as well as the differences in levels of depressiveness and in broken stick patterns between cohort strata. These analyses were adjusted for gender and parents’ occupational status, classified as white collar, blue collar, or mixed collar.

## Results

The cohort consisted of 1,001 individuals, comprising 482 women and 519 men. Among them, 287 individuals hailed from a blue-collar, 347 from a mixed-collar, and 367 from a white-collar family.

**Figure 1:**
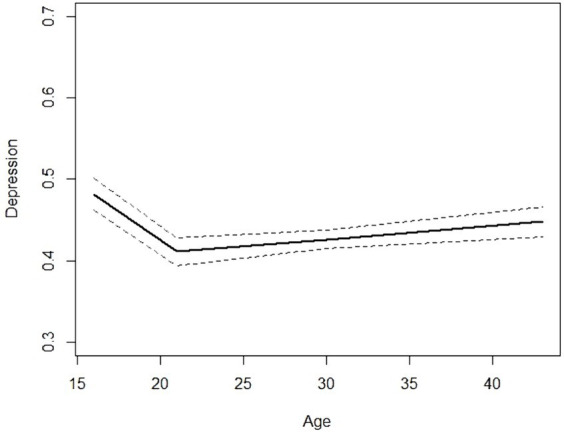
‘Broken stick’ model of the development depression symptom score with 95% confidence interval of the Northern Swedish Cohort from teenage to middle age.

Figure 1 illustrates the broken stick pattern in the development of depression symptom scores within the total cohort. The broken stick model was found to be significantly different from the linear model (*p* < 0.0001).

**Figure 2:**
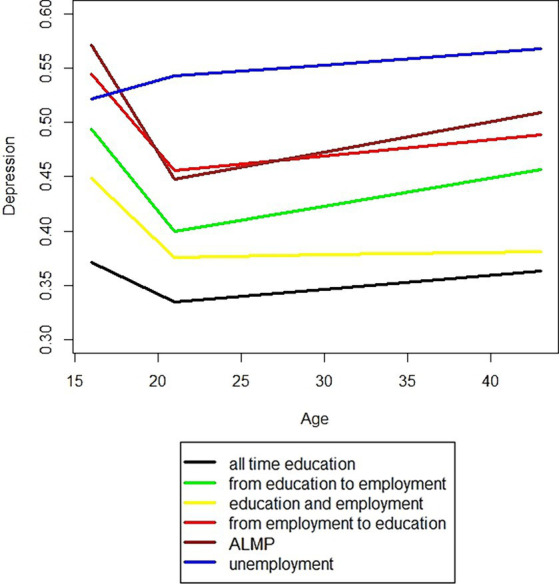
Depression symptom score of the Northern Swedish Cohort from teenage to middle age by type of labour market attachment from age 18 to age 21. ALMP: participation in ‘Active Labour Market Policy’ program.

When we analysed the data by ILMA categories (Figure 2), significant differences in the broken stick pattern were observed (*p* = 0.0218). The most pronounced patterns were observed in the ‘from education to employment’ and ‘ALMP’ groups, with a less pronounced angle in the ‘all-time education’ group. In the ‘unemployment’ group, depressiveness appeared to increase linearly.

There were clear differences in the levels of depressive symptoms between the six groups (*p* < 0.0001), with the lowest level observed in the ‘all-time education’ group and the highest in the ‘unemployment’ group.

In sum, the anticipated relationship between a firm Initial Labour Market Attachment (ILMA) and a reduction in depressiveness received support in three out of four groups classified as ‘firm.’ The deviation from this pattern in the ‘all-time education’ group does not contradict our expectations because their depressiveness was already quite low during school age, leaving little room for further reduction.

It’s also not surprising that there’s virtually no ‘broken stick’ pattern in the case of ‘unemployment’, as previous research has demonstrated its negative impact on mental health (9, 24, 27). However, the steep decrease in depressiveness in the ‘ALMP’ group contradicts our expectations. This finding is intriguing because enrolment in policy intervention programs often implies concerns in ILMA.

The status trajectories within the ‘ALMP’ group indicate that individuals participate in policy measures but also experience bouts of unemployment, particularly at the beginning and end, while also securing some employment. For the ‘unemployment’ group, it’s evident that in Sweden persistent unemployment among young people is not continual; rather, it is interspersed with periods of employment, education, and engagement in policy measures. Notably, employment trajectories in both the ‘ALMP’ and ‘unemployment’ groups are similar in both their level and pattern. Furthermore, both groups are of equal size (n = 98 and n = 118, respectively). What, then, determines an individual’s belonging to one group over the other?

## Discussion

During the 1980s, all unemployed young people were intended to be offered ALMP. However, due to the insufficient capacity or volume of these activities, some individuals remained or became unemployed. Consequently, an individual’s placement into ALMP was largely influenced by chance. Within this context, ALMP appears to function as an intervention that not only improves the short-term mental health of participants but also exhibits long-term benefits compared to those enduring persistent unemployment.

In general, depressive symptoms among ALMP participants exhibit a decreasing pattern like that of the four groups that seem to secure firm attachment soon after completing their basic education. This may indicate that improvement of their mental health is largely by virtue of psychosocial functions similar those provided by regular employment or conventional studying These functions may include financial compensation, social contacts, establishment of social status and personal identity, time structure of the day, and an opportunity to strive towards collective purposes and shared experiences (32). This appears to hold true even within the relatively small group that represents a counter-mainstream development, transitioning ‘from employment to education’. The fact that this particular group shows the highest level of depression initially and sustains a relatively high level later on suggests that their depression may primarily stem from factors other than labour market attachment.

In terms of depression levels, the findings align with a previous study on NoSCo, indicating that youth exposure to unemployment predicts long-term ‘scars’ in mental health extending into middle age, especially when compared to exposure to ALMP (33).

Additionally, significant differences by ILMA were observed not only in the main outcome, namely the ‘broken stick’ angle, but also in the level of the curves illustrating the development of depressiveness. This finding aligns with earlier evidence that the association between unemployment and reduced mental well-being appears to be pronounced during young age (9).

This study provides evidence supporting the concept of emerging adulthood as a pivotal stage in the life course with significant implications for mental health development. These findings hold true even when adjusting for potential confounders such as gender and parental social class. Specifically, the study contributes novel insights to the existing body of research (34) that demonstrates the beneficial effects of ALMP on mental health. Future research should address whether ALMP, applied in older ages or different contexts, also contribute to improved health outcomes. Moreover, it might be interesting to study the interactions between ILMA and mental health on labor market attachment during subsequent life course of the NoSCo. Regarding NEET individuals, the trajectory approach could help disentangle the dynamics of the labor market attachment among those who do not transit from school to work in the mainstream. Finally, it’s essential to recognize that while ILMA is a crucial developmental task during emerging adulthood, it is just one of several challenges individuals may face during this period.

### Methodological aspects

The measure of depressiveness in NoSCo was derived from items included in the baseline survey at age 16 in 1981. These items were consistently worded in the follow-up surveys. While specific to the cohort, this measure can be considered a valid indicator of depressiveness even in repeated surveys as respondents age and their life contexts and conditions change (28). Therefore, a high level of the variable at age 16 is not attributable to age-related bias; rather, it reflects mature depressiveness rather than the emotional aftermath of puberty or transient emotional turmoil experienced by teenagers.

Multiple response trajectory analysis has emerged as a preferred method for investigating longitudinal data. However, it has been rarely applied in the study of labour market attachment. It is particularly well-suited for researching the attachment during young age, when there is a wide range of potential statuses and frequent status transitions.

The analysis reveals a linear development of depressiveness from age 21 to 43. As a form of sensitivity analysis, we also examined age 30 for any potential ‘broken stick’ pattern, but a linear trend appeared to better fit the development at that point in the life course. The slightly upward and relatively parallel diretions of the lines indicate that the mental well-being of the NoSCo is generally declining rather than improving across all ILMA groups up to middle age.

## Conclusion

In terms of policy recommendations, the consistently high levels of depressiveness within the ‘unemployment’ group advocate for the need to reduce long-term and repeated unemployment in young age. The findings related to ALMP provide evidence of the intervention’s benefits. While the primary goal of these measures is to strengthen the LMA, they also incorporate elements that promote participants’ mental health. ALMP comprises various measures, and the NoSCo data does not allow for studying them separately concerning mental health. Therefore, it is not possible to provide specific recommendations regarding contents of ALMP for today’s policymakers. Nonetheless, it remains important to adhere to the principles of universalism, comparatively generous replacement rates, and extensive welfare services.

Even though this study pertains to the general population with very low attrition in the sample during the follow-up, it is advisable to conduct replications to determine whether the findings are exceptional and cohort-specific, specific to (Northern) Sweden, or if similar patterns of development and associations with depressiveness occur in countries with various welfare services. This is especially relevant to the notable policy implication that ALMP has a positive effect: it seems to reach participants with relatively poor baseline mental health, and their mental well-being improves. This finding suggests promise that employment policy interventions in adulthood or in other historical contexts may yield similar effects.

## Data availability statement

The raw data supporting the conclusions of this article will be made available by the authors, without undue reservation.

## Ethics statement

The studies involving humans were approved by The Regional Ethics Vetting Board in Umeå, Sweden. The studies were conducted in accordance with the local legislation and institutional requirements. Written informed consent for participation was not required from the participants or the participants' legal guardians/next of kin because According to the local legislation and institutional requirements the consent was not required at the time of starting the follow-up study in 1981.

## Author contributions

PV: Conceptualization, Formal analysis, Methodology, Writing – original draft. TN: Formal analysis, Methodology, Writing – original draft. HW: Writing – review & editing. P-OÖ: Writing – review & editing. UJ: Data curation, Investigation, Writing – original draft. AH: Funding acquisition, Investigation, Project administration, Writing – original draft.
