# Supplementary material for: Active labour market policies in emerging adulthood may act as a protective factor against future depressiveness: an analysis of the long-term trajectories of depressive symptoms in the Northern Swedish Cohort
Source: Front Public Health. 2024 Apr 9;12:1345034. doi: 10.3389/fpubh.2024.1345034 (PMC11035740; doi:10.3389/fpubh.2024.1345034)
Supplement: Supplementary file 1 [file Data_Sheet_1.PDF]

Additional file:

Figures of the nine latent classes of the labour market attachment from age 18 to age 21 of the Northern Swedish Cohort, and disposition of the classes into six groups.

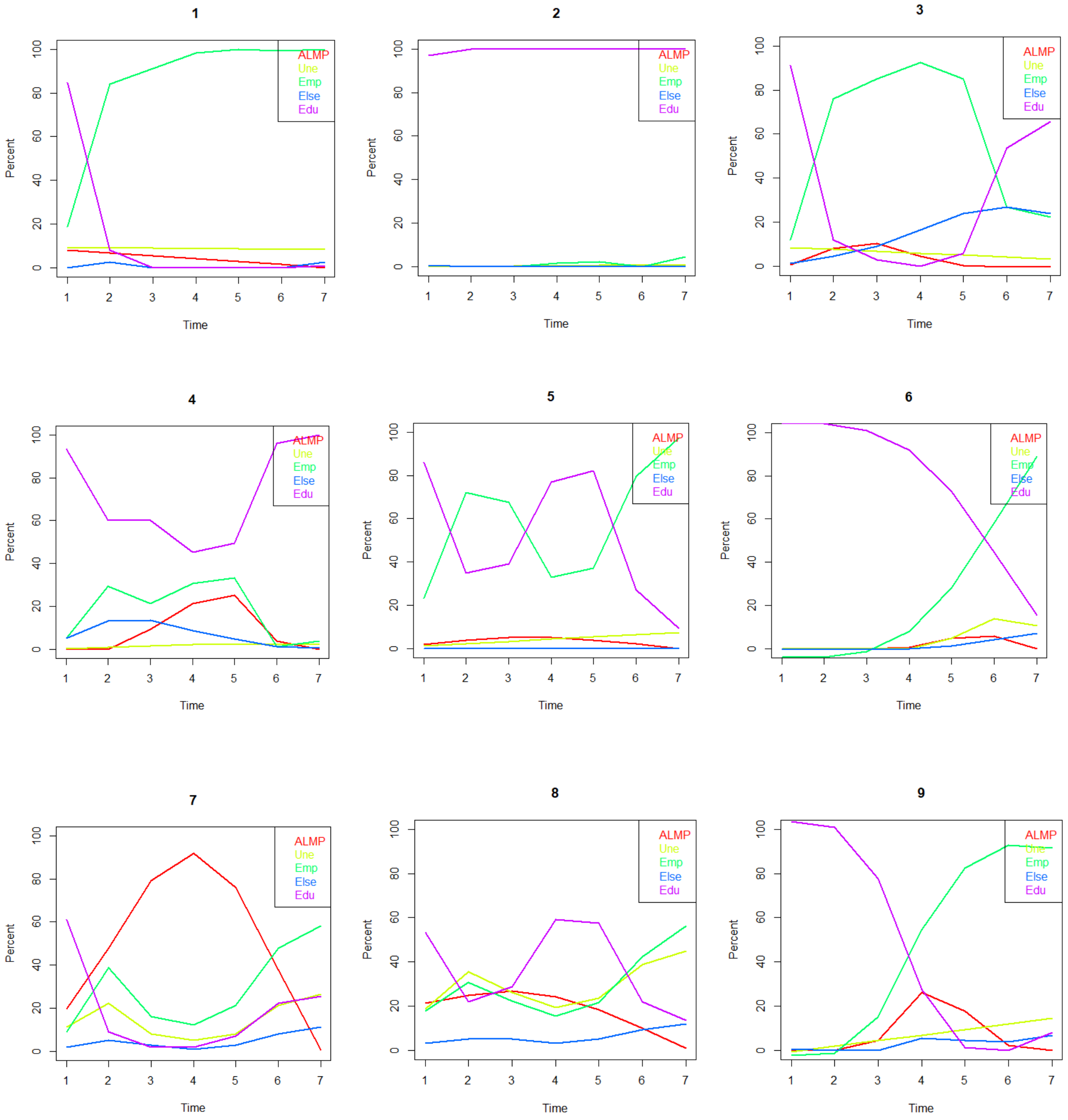

Group 1. All-time education' (Class 2, n=129) - individuals who were continuously studying throughout the time window.

Group 2. 'From education to employment' (Classes 1, 6, and 9, n=363) - individuals whose school-to-work transition took place within the time window.

Group 3. 'Education and employment' (Classes 4 and 5, n=226) - individuals who worked and studied simultaneously.

Group 4. 'From employment to education' (Class 3, n=67) - individuals who experienced a reverse 'work-to-school' transition.

Group 5. 'Active Labor Market Policy' (ALMP) (Class 7, n=98) - individuals who participated in ALMP programs during the time window.

Group 6. 'Unemployment' (Class 8, n=118) - individuals characterized by a high level of unemployment, although they also worked, studied, and participated in ALMP programs.
